# A Novel Multispace Image Reconstruction Method for Pathological Image Classification Based on Structural Information

**DOI:** 10.1155/2019/3530903

**Published:** 2019-04-11

**Authors:** Honglin Zhu, Huiyan Jiang, Siqi Li, Haoming Li, Yan Pei

**Affiliations:** ^1^Department of Software College, Northeastern University, Shenyang 110819, China; ^2^School of Computer Science and Engineering, the University of Aizu, Aizuwakamatsu 965-8580, Japan

## Abstract

Pathological image classification is of great importance in various biomedical applications, such as for lesion detection, cancer subtype identification, and pathological grading. To this end, this paper proposed a novel classification framework using the multispace image reconstruction inputs and the transfer learning technology. Specifically, a multispace image reconstruction method was first developed to generate a new image containing three channels composed of gradient, gray level cooccurrence matrix (GLCM) and local binary pattern (LBP) spaces, respectively. Then, the pretrained VGG-16 net was utilized to extract the high-level semantic features of original images (RGB) and reconstructed images. Subsequently, the long short-term memory (LSTM) layer was used for feature selection and refinement while increasing its discrimination capability. Finally, the classification task was performed via the softmax classifier. Our framework was evaluated on a publicly available microscopy image dataset of IICBU malignant lymphoma. Experimental results demonstrated the performance advantages of our proposed classification framework by comparing with the related works.

## 1. Introduction

Pathological image classification is important in various biomedical applications, such as for lesion detection [1], cancer subtype identification [2], and pathological grading [3]. However, it is difficult for human eyes to recognize subtle differences in the tissue; there are thus different interpretations among medical experts. Moreover, pathologists generally perform the pathological image classification by the microscopic examination, which is time-consuming, operator intensive and subjective. Therefore, computer-aided diagnosis (CAD) is indispensable for pathological image analysis.

Traditionally, machine learning (ML) techniques have been widely used for different medical image processing tasks, including image detection [4], segmentation [5], and recognition [6]. Specifically, many researchers applied them to the field of pathological image analysis, which has achieved a great performance. For example, an automated system [7] was used to extract a set of texture features by multiwavelets, Gabor-filters, gray level cooccurrence matrix (GLCM), and fractal dimensions (FD) for grading pathological images of prostatic carcinoma. Experimental results showed that FD-based features set had very good performance and provided useful information for pathological image classification. If FD-based features were included in the feature set and optimized, the classification accuracy can be increased to 95.6%. Jia et al. [8] proposed an unsupervised network for image segmentation by learning the nonlinear distribution of medical data without prior knowledge. Experimental results demonstrated that the unsupervised one-class support vector machine (SVM) had better segmentation results than a supervised two-class SVM. To sum up, conventional machine learning methods are generally performed based on discriminative hand-craft features from manual features, but the abstract level of the manual features is relatively low, which brings difficulties for subsequent experiments.

Recently, several CAD models based on deep learning (DL) strategies have been developed for pathological image processing, which achieve the automatic extraction of features and improve the accuracy of classification. Hence, CAD using deep learning significantly reduces the subjective misjudgment of doctors. For instance, Sirinukunwattana et al. [9] explored the effect of the convolutional network depth on its accuracy in the localization and classification track respectively. Xu et al. [10] employed a deep convolutional neural network (CNN) to learn the high-level representations of histopathology images based on the transfer learning technology. In other words, initial weights of the model were determined using the optimal parameters pretrained on ImageNet. Lei et al. [11] accomplished a work with respect to a weakly supervised classification and disease localization using pretrained deep convolutional network. Gu et al. [12] employed the classical AlexNet to analyze the corresponding classification problems. The classification accuracy obtained by the proposed model can achieve to 69.9% in the task of cancer classification. Maga$\tilde{n}$a-Tellez et al. [13] proposed a spatially constrained convolutional neural network (SC-CNN) to perform nucleus detection and develop a novel neighboring ensemble predictor for classification of nuclei. Mercan et al. [14] proposed a deep learning method, which used multiple processing layers to learn representations of data through multiple levels of abstraction. Compared to traditional ML methods, DL can learn low-level features as well as high-level semantic information. Moreover, the extracted features have a stronger generalization performance than the specific settings and can be applied to multiple fields.

Generally, DL methods require a large number of training data. However, one challenge is how to deal with the lack of data samples in the field of medical images. For example, Shang et al. [15] proposed a novel algorithm that can preserve geometric structure information based on the feature selection framework of subspace learning and then used the *L*_2,1_-norm to ensure the sparsity of the feature array and avoid complicated solutions. However, it was expensive and impossible to train an effective model using a small amount of labeled data. To address this issue, the transfer learning technique had been used in this framework, which improved model performance. Gupta et al. [16] proposed a heterogeneous transfer learning framework, which extracted textual information to achieve the image classification. Experimental results showed that this framework performed better than other methods under the premise of using the less labeled data.

As previously mentioned, features extracted by DL and transfer learning are beneficial for pathological image classification. However, different features represent different image characteristics; there are thus many literatures to obtain more comprehensive information via image feature fusion. For instance, Li et al. [17] proposed a multimodal feature fusion-based framework to achieve representations of geographic images by leveraging a low-to-high learning flow for both the deep and shallow modality features. Banerjee et al. [18] presented deep-learning-based CADs for the diagnosis of subtypes of rhabdomyosarcoma (RMS) by analyzing multiparametric MR images. They achieved creating a comprehensive representation of tumor by a fusion method. Finally, they used a pretrained deep convolutional neural network to perform classification of two RMS subtypes, which achieved a fast, efficient, and reproducible diagnosis for RMS subtypes.

In this study, we propose a pathological image classification framework based on a multispace image reconstruction method and the transfer learning technology. The contributions of this paper are summarized as follows. First, the reconstructed image is a new generated image containing three channels composed of gradient, gray level cooccurrence matrix (GLCM) and local binary pattern (LBP) spaces. Specifically, the gradient image is sensitive to the boundaries and GLCM image is sensitive to regions of nuclei. The LBP image mainly highlights the center of each nucleus. Second, high-level semantic features from RGB and pseudocolor images are extracted via the pretrained VGG-16 net and the long short-term memory (LSTM) layer is used to reduce the feature dimension while increasing its discrimination capability. Our framework is evaluated using a publicly available microscopy image dataset of IICBU malignant lymphoma [19]. Experimental results demonstrate the performance advantages of our proposed classification framework by comparing with the related works.

The remainder of this paper is organized as follows. In Section 2, we introduce NHL pathological image dataset and our classification framework.  Section 3 shows the classification results and a comprehensive comparison with some other methods. Finally, discussions and conclusions are summarized in Sections 4 and 5, respectively.

## 2. Materials and Methods

### 2.1. Dataset Description

The IICBU lymphoma dataset contains 374 hematoxylin and eosin (H&E) stained non-Hodgkin lymphoma (NHL) images, which are divided into three classes: chronic lymphocytic leukemia (CLL), follicular lymphoma (FL), and mantle cell lymphoma (MCL). They are the major types of malignant small B-cell lymphoma [19]. The dataset sections are captured using the bright field microscopy and each image is 1388 × 1040 pixels. In our experiments, each image is divided into 336 nonoverlapped patches with 64 × 64 pixels (21 × 16); we perform the classification tasks in the patch level and image level, and the image classification results are determined via the classification results of the corresponding patches.

### 2.2. Pathological Image Classification

**Figure 1:**
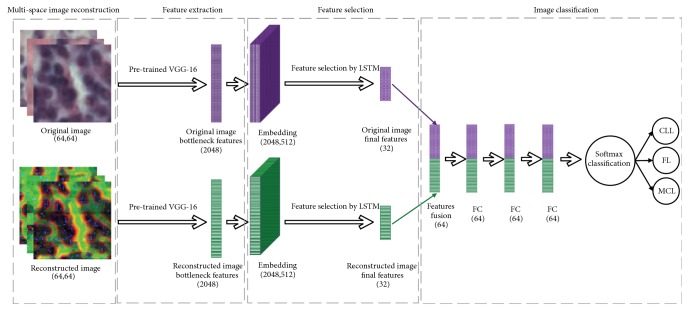
The flowchart of the proposed classification process, which has four steps. (1) Multispace image reconstruction; (2) feature extraction; (3) feature selection; and (4) image classification.

As shown in Figure 1, the proposed process of the pathological image classification is described, which is summarized as four stages, including multispace image reconstruction, feature extraction, feature selection, and image classification. First, a multispace image reconstruction method is developed to generate new color images, which contain the gradient, GLCM, and LBP information adequately. Second, the VGG-16 net pretrained on ImageNet is applied to original images and reconstructed images for feature extraction. Third, the long short-term memory (LSTM) layer is used for feature selection. Finally, the softmax classifier is utilized to perform the classification task.

#### 2.2.1. Multispace Image Reconstruction

**Figure 2:**
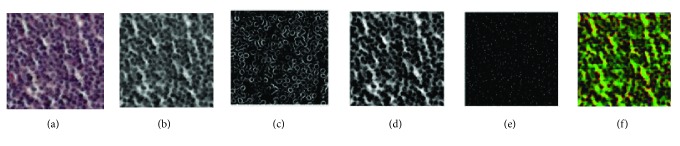
The process of multispace image reconstruction. (a) Original image. (b) Grayscale image. (c) Gradient image. (d) GLCM. (e) LBP. (f) Our reconstructed image.

Generally, H&E images vary significantly in color, because of many factors, including specimen preparation and staining protocol inconsistencies (e.g., temperature of solutions); variations in fixation characteristics and interpatient variation and the scanner are used to digitize the slides. The classification performance could be hampered by color and intensity variations. Many classification tasks implemented by DL strategies not only considered RGB inputs but also took the advantage of other spaces' inputs, such as HSV, Lab, and YUV [20]. Inspired by these works, we first use the white balance method to alleviate color influence and then convert RGB images into grayscale images. Besides, in order to consider more beneficial information, we propose a novel multispace reconstruction method to generate a new image which is composed of the gradient, GLCM and LBP spaces' images. In other words, the R, G, and B channels of a reconstructed image are represented as the gradient, GLCM, and LBP spaces' images, respectively.  Figure 2 presents the generative process of a reconstructed image.  Figure 2(a) is an original H&E pathological image. Figures 2(b)–2(e) represent the grayscale, gradient, GLCM, and LBP images.  Figure 2(f) is the generated reconstructed image. Visually, the gradient image is sensitive to boundaries and the GLCM image is sensitive to the regions of nuclei. The LBP image mainly emphasizes the center of each nucleus. The following experiments demonstrate that these three kinds of information are effective for the pathological image classification.

#### 2.2.2. Feature Extraction via VGG-16 Net

**Figure 3:**
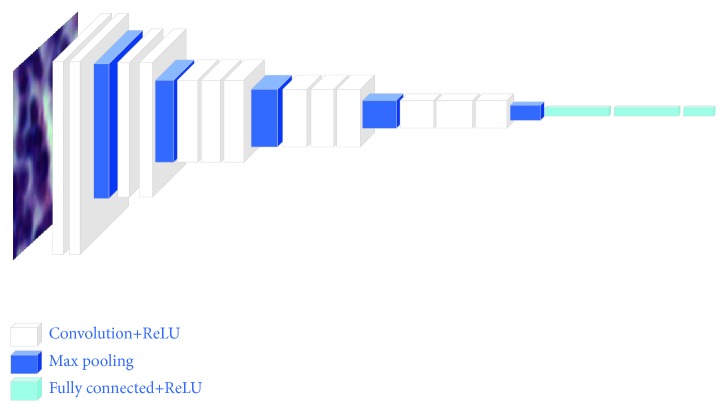
The architecture of the VGG-16.

As is well known, the effective classification greatly depends on the discriminative representations of samples. Some literatures [21] treated the feature extraction and classifier design as two separated processes; they could not work together to maximally extract and retain the most discriminative information. Recently, deep learning strategies are widely applied to the feature extraction on different image classification tasks. Particularly, conventional convolutional neural network (CNN) [22] is consisted of alternating convolution and subsampling operations. Flatten operation was used on the feature maps of the last convolution layer to obtain the feature vectors. The next several fully connected layers were applied one by one on the feature vectors. These series of operations were regarded as a process of feature extraction. In addition, the transfer learning in [23] used the parameters of trained models (via natural images) to extract medical images' features or initialized the parameters of a particular model. Motivated by [24], our strategy uses the pretrained VGG-16 [25] (Visual Geometry Group) net for performing the feature extraction on lymphoma patches. VGG-16 is a simple and common deep convolutional neural network and can obtain the competitive performances with other networks. The proposed feature extraction process using VGG-16 net is described in Figure 3. The network is trained on the entire ImageNet [26] dataset and our input images are all resized to 64×64 pixels. The corresponding outputs are 2048-dimensional features. The specific network structure for our classification task is presented in Table 1. Note that the pretrained VGG-16 net is used for the feature extraction on RGB images and the reconstructed patches simultaneously.

#### 2.2.3. Feature Selection by LSTM

Due to the great differences between natural images and pathological images, the features extracted by pretrained VGG-16 net may not obtain the satisfactory classification results. A common method for addressing this issue is to initialize the model's parameters using the pretrained net and then train our images to adjust the parameters. However, there is no essential difference compared with training own data directly, and it also could lead to the overfitting. To this end, we propose a “feature selection strategy” to find the discriminative features and remove the redundant features using the long short-term memory (LSTM) layer. The LSTM [27] is an efficient network for text classification tasks, which considers the relationship between the text and time information. For our case, the relationship between original RGB images and corresponding reconstructed images is similar to temporal text data. Specifically, redundant features are removed via the forget gate, then beneficial features are added to the input gate and the optimal feature vector is obtained by the output gate. Figure 1 shows the feature selection process using the LSTM layer. The VGG-16 features of RGB images are regarded as the original content information (text information in LSTM), while the VGG-16 features of the corresponding reconstructed images are considered as the auxiliary information (temporal information in LSTM). There are 32-dimension features selected via the LSTM layer. Afterwards, we cascade the 32-dimension features of RGB images and the reconstructed images into a 64-dimension feature vector. Finally, the softmax classifier is used to perform the pathological image classification task. Note that the integral loss function is defined as follows in (1)-(3).

(1) Loss=Lossoriginal+Lossreconstruction

(2) Lossoriginal=−∑iyilog⁡ai

(3) Lossreconstruction=−∑iyilog⁡bi

where *y*_*i*_ represents the label of each sample. *a*_*i*_ and *b*_*i*_ are the output probabilities obtained via the softmax function *e*^*z*_*i*_^/∑_*k*_*e*^*z*_*k*_^, *z*_*i*_ = ∑_*j*_*w*_*ij*_*x*_*ij*_ + *b* is the output of the neuron, *x*_*ij*_ represents the feature vectors of the RGB and reconstructed inputs, respectively, and *w*_*ij*_ and *b* are the corresponding weights and bias. We can observe that if one loss function (*Loss*_*original*_ or *Loss*_*reconstruction*_) leads to the vanishing gradient, the feedback information from another loss function can also train the LSTM layer. In order to remove redundant information in the features, the LSTM layer for text classification tasks is used in this paper by analyzing each row of the feature matrix recursively, and it has produced good results.

## 3. Results and Comparisons

### 3.1. Experimental Results

In order to verify the effectiveness of the proposed framework, three commonly used evaluative criteria are considered as follows in (4)-(6).

(4) ACC=1n∑i=1nIfxi=yi

(5) SENci=NumPTciNumGTci×100%

(6) SPEci=NumPT−ciNumGT−ci×100%

where ACC means the overall grading accuracy. SEN(*c*_*i*_) and SPE(*c*_*i*_) are sensitive and specificity for each class (*c*_*i*_). I(*f*(*x*_*i*_) = *y*_*i*_) is the indicative function. It defines that if *f*(*x*_*i*_) = *y*, I(*f*(*x*_*i*_) = *y*_*i*_) = 1; otherwise I(*f*(*x*_*i*_) = *y*_*i*_) = 0. −*c*_*i*_ denote all the other classes except (*c*_*i*_). Num(PT(*c*_*i*_)) and Num(GT(*c*_*i*_)) denote the number of correctly predicted class (*c*_*i*_) and the total number of (*c*_*i*_) in the ground truth, respectively.

**Figure 4:**
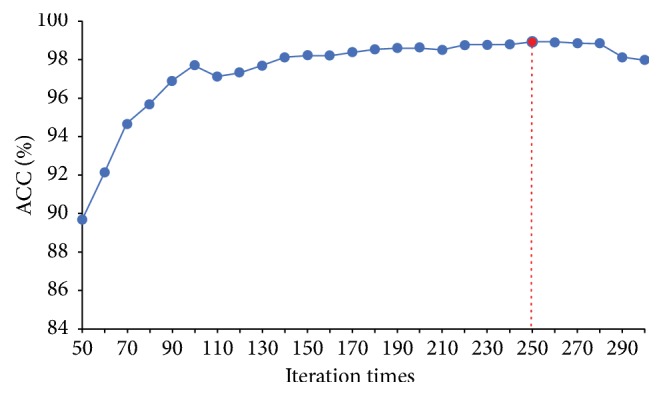
Accuracy versus iteration times graph.

In our experiments, first, we perform the NHL classification tasks using the VGG-16 features (Section 2.2). Our experiments adopt 10-cross validation method to determine the optimal parameters of each model.  Table 2 presents the different classification results with the VGG-16 features extracted on the original and reconstructed images. We can observe that the feature combination (4096-dimension) is beneficial for our classification task. It can be also proved that reconstructed images can provide complementary information with respect to RGB images. As is well known, more features could not denote that they can achieve a better result. This is because that there are redundant features which can result in the worse classification results. To address this issue, the feature selection strategy is widely used for the more accurate classification result while reducing the computational cost.  Table 3 shows different classification results using different features selected by the LSTM layer. It can be observed that the LSTM layer is effective for feature selection and improving classification accuracy. Following these experimental results, our proposed framework for NHL image classification is determined. In addition, Figure 4 shows the classification results according to different iteration times and the best time is 250.

### 3.2. Image-Level Classification

**Figure 5:**
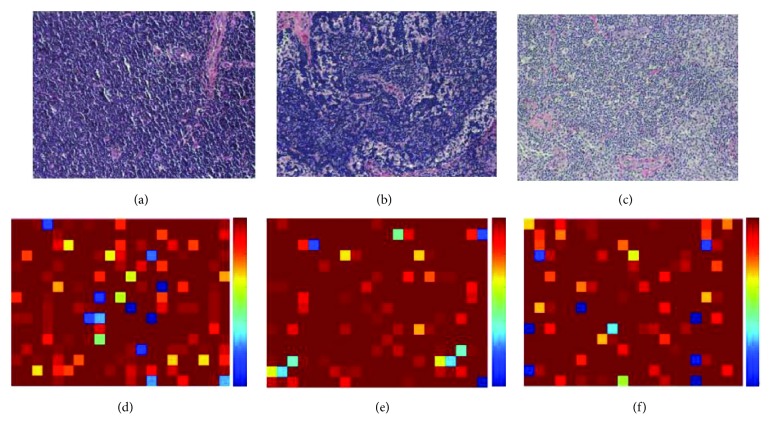
The probability atlases of three examples with different classes. (a) A CLL pathological image. (b) A FL pathological image. (c) An MCL pathological image. (d–f) The probability atlas of (a–c), respectively.

As mentioned before, our objective of this paper is to classify 374 NHL pathological images, which include CLL, FL, and MCL. Our proposed classification framework is performed on the image patches with 64×64×3; the image-level classification is thus accomplished via the label of each patch inside a whole pathological image. Specifically, a pathological image is divided into 336 nonoverlapped patches and the label of each patch is obtained via the proposed classification framework. We can thus determine the pathological image's label using the majority voting strategy via the number of each category's patches. To visualize the classification results of the whole pathological images, Figure 5 shows the probability atlases of three examples with different classes. Figures 5(a)–5(c) are CLL, FL, and MCL pathological images. Figures 5(d)–5(f) are corresponding probability atlases. The colors in the probability atlases represent the probability that each patch is predicted to be a label of the image categories. The colors from bottom to top is ranged from [0,1]. According to the probability atlases, we can see that majority of patches inside a pathological image are correctly classified as the corresponding image's label.

### 3.3. Comparisons

**Figure 6:**
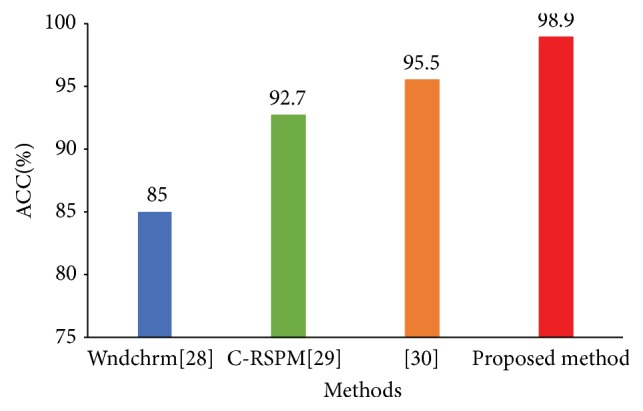
The average classification accuracy (%) using different methods.

To further evaluate our proposed classification framework with other models, there are three popular methods [28–30] used for comparison in our experiments. Shamir et al. [28] developed CAD software for biological image analysis. This software worked by first extracting image content descriptors from the raw images, image transforms, and compound image transforms. Then, the most informative features were selected and then used for classification and similarity measurement. Meng et al. [29] proposed a framework using the novel and robust collateral representative subspace projection modeling (CRSPM) supervised classification model for general histology image classification. Codella et al. [30] first created additional 5 images for each H&E pathological image, which can emphasize unique aspects of the original image, such as dominant staining and staining segmentations. Then a pretrained CNN model was used to extract 4096-dimension visual features. Finally, nonlinear SVMs were utilized to perform the classification. Similarly, all methods are performed on NHL dataset and tested using 10-cross validation method. The average comparison results are presented in Figure 6. We can observe that our method performs the best in terms of ACC.

## 4. Discussions

According to the classification results in Section 3.1 (Tables 2 and 3), there are two important characteristics with respect to our proposed classification framework. First, besides RGB image information, we also consider the other classes of information via a “multispace image reconstruction” strategy. As Figure 2 is shown, the gradient image is sensitive to boundaries and GLCM image is sensitive to regions of nuclei. The LBP image mainly highlights the center of each nucleus. The results indicate that this auxiliary information facilitates the improvement of classification accuracy significantly. Second, in order to remove redundant features, the pretrained LSTM layer is used to perform a “feature selection” process. Further, our classification framework obtains the ACC of 98.94% in patch-level classification.

Comparing results from our classification framework with other methods, the software proposed in [28] only used hand-craft features to perform classification tasks and considered no high-level semantic features (extracted via deep learning model). Our method and [29] both converted the image-level classification into the patch-level classification. A difference from [29] was that it divided the image into 25 overlapped patches with a larger size. This, however, may lose more subtle local information. Finally, the method in [30] utilized a pretrained CNN to extract the visual features from different images. Our improvement related to [30] is that we adopt a feature selection method to increase its discrimination capability. The results in Figure 6 demonstrate the advantages of our proposed classification framework.

## 5. Conclusions

In this paper, we proposed a novel classification framework based on a multispace image reconstruction method and the transfer learning technology. The multispace image reconstruction mapping method can convert original RGB images into gradient, gray level cooccurrence matrix (GLCM), and local binary pattern (LBP) spaces. This auxiliary information is beneficial for more accurate classification results. Then, the pretrained VGG-16 net was utilized to extract the high-level semantic features of RGB and reconstructed images. Subsequently, the LSTM layer was used for feature selection and refinement while increasing its discrimination capability. Experimental results demonstrated the performance advantages of our proposed classification framework by comparing with related works. Future work may be considered based on our proposed framework. For feature extraction, we plan to establish a complete feature set, which includes hand-craft features and high-level representations. Another important work will consider the feature extraction on more other spaces' images.

## Figures and Tables

**Table 1:** The network structure for our classification task.

Layer	Filter number	Kernel size	Dimension
Input	-	-	64 × 64 × 3
Conv1	64	3 × 3	64 × 64 × 64
Conv2	64	3 × 3	64 × 64 × 64
Max-Pool1	-	2 × 2	32 × 32 × 64
Conv3	128	3 × 3	32 × 32 × 128
Conv4	128	3 × 3	32 × 32 × 128
Max-Pool2	-	2 × 2	16 × 16 × 128
Conv5	256	3 × 3	16 × 16 × 256
Conv6	256	3 × 3	16 × 16 × 256
Conv7	256	3 × 3	16 × 16 × 256
Max-Pool3	-	2 × 2	8 × 8 × 256
Conv8	512	3 × 3	8 × 8 × 512
Conv9	512	3 × 3	8 × 8 × 512
Conv10	512	3 × 3	8 × 8 × 256
Max-Pool4	-	2 × 2	4 × 4 × 256
Conv11	512	3 × 3	4 × 4 × 512
Conv12	512	3 × 3	4 × 4 × 512
Conv13	512	3 × 3	4 × 4 × 512
Max-Pool5	-	2 × 2	2 × 2 × 512

**Table 2:** Different classification results using RGB (2048-dimension), reconstruction (2048-dimension), and combination features (4096-dimension).

Index	Classes	RGB	Reconstruction	Combination
ACC	Overall	(0.5317 ± 0.016)	(0.4423 ± 0.016)	(0.6667 ± 0.008)

	CLL	(0.5205 ± 0.011)	(0.4214 ± 0.010)	(0.6414 ± 0.010)
SEN	FL	(0.5179 ± 0.010)	(0.4187 ± 0.013)	(0.6396 ± 0.018)
	MCL	(0.5224 ± 0.009)	(0.4226 ± 0.009)	(0.6428 ± 0.009)

	CLL	(0.5515 ± 0.008)	(0.4602 ± 0.004)	(0.6806 ± 0.005)
SPE	FL	(0.5469 ± 0.012)	(0.4584 ± 0.011)	(0.6789 ± 0.007)
	MCL	(0.5524 ± 0.011)	(0.4628 ± 0.014)	(0.6833 ± 0.009)

**Table 3:** Different classification results using RGB (32-dimension), reconstruction (32-dimension), and combination features selected by the LSTM layer (64-dimension).

Index	Classes	RGB-LSTM	Reconstruction-LSTM	Combination-LSTM
ACC	Overall	(0.8453 ± 0.018)	(0.7637 ± 0.012)	(0.9894 ± 0.011)

	CLL	(0.8243 ± 0.014)	(0.7459 ± 0.019)	(0.9666 ± 0.012)
SEN	FL	(0.8168 ± 0.010)	(0.7321 ± 0.009)	(0.9662 ± 0.011)
	MCL	(0.8251 ± 0.019)	(0.7482 ± 0.015)	(0.9685 ± 0.013)

	CLL	(0.8721 ± 0.014)	(0.7832 ± 0.008)	(0.9931 ± 0.007)
SPE	FL	(0.8659 ± 0.011)	(0.7779 ± 0.007)	(0.9912 ± 0.005)
	MCL	(0.8718 ± 0.012)	(0.7834 ± 0.011)	(0.9938 ± 0.013)

## Data Availability

(i) The dataset includes three types of malignant lymphoma. (ii) The data can be accessed from https://ome.grc.nia.nih.gov/iicbu2008/lymphoma/index.html. In addition, there are no restrictions on data access.
